# Role of pyroptosis-related cytokines in the prediction of lung cancer

**DOI:** 10.1016/j.heliyon.2024.e31399

**Published:** 2024-05-15

**Authors:** Zhouyangfan Peng, Xiqing Tan, Yang Xi, Zi Chen, Yapei Li

**Affiliations:** a Health Management Center, The Third Xiangya Hospital, Central South University, Changsha, 410013, China; b Department of General Practice, The Third Xiangya Hospital, Central South University, Changsha, 410013, China; c Department of Pharmacy, The Second Xiangya Hospital, Central South University, Changsha, 410011, China

**Keywords:** Pyroptosis-related cytokines, Lung carcinoma, Biomarkers, Diagnosis

## Abstract

**Objectives:**

Lung cancer is the leading cause to induce cancer-related mortality. Effective biomarkers for prediction the occurrence of lung cancer is urgently needed. Our previous studies indicated that pyroptosis-related cytokines TNF-α, IFN-γ, MIP-1α, MIP-1β, MIP-2 and IP-10 is important to influence the efficacy of chemotherapy drug in lung cancer tissues. But the role of pyroptosis-related cytokines in prediction the occurrence of lung cancer is still unknown.

**Methods:**

Blood samples were collected from 258 lung cancer patients at different stage and 80 healthy volunteers. Serum levels of pyroptosis-related cytokines including TNF-α, IFN-γ, MIP-1α, MIP-1β, MIP-2 and IP-10 were measured by Cytometric Bead Array (CBA). ROC curve was performed to evaluate the cut-off value and diagnosis value for prediction and diagnosis of lung cancer.

**Results:**

Compared with control group, the levels of IP-10, MIP-1α, MIP-1β, MIP-2 and TNF-α were significantly higher in lung cancer patients (45.5 (37.1–56.7): 57.2 (43.0–76.5), 34.4 (21.8–75.2): 115.4 (96.6–191.2), 49.3 (25.6–78.7): 160.5 (124.9–218.6), 22.6 (17.8–31.2): 77.9 (50.1–186.5), 3.80 (2.3–6.2): 10.3 (5.7–16.6)), but the level of IFN-γ was decreased in the patients (12.38 (9.1–27.8): 5.9 (3.5–9.7)). All the above cytokines were significantly associated with the diagnosis of lung cancer, and the AUC values of IFN-γ, IP-10, MIP-1α, MIP-1β, MIP-2, and TNF-α were 0.800, 0.656, 0.905, 0.921, 0.914, and 0.824. And the AUC can rise to 0.986 after combining the above factors, and the sensitivity and specificity also up to 96.7 % and 93.7 %, respectively. Additionally, TNF-α (r = 0.400, P < 0.01), MIP-2 (r = 0.343, P < 0.01), MIP-1α (r = 0.551, P < 0.01) and MIP-1β (r = 0.403, p < 0.01) were positively associated with occurrence of lung cancer, but IFN-γ (r = −0.483, p < 0.01) was negatively associated with occurrence of lung cancer. As far as the potential of early diagnosis of lung cancer, TNF-α (AUC = 0.577), MIP-1α (AUC = 0.804) and MIP-1β (AUC = 0.791) can predict the early stage of lung cancer, and combination of the above three cytokines has a better predictive efficiency (AUC = 0.854).

**Conclusion:**

Our study establishes a link between the levels of IP-10, MIP-1α, MIP-1β, MIP-2, TNF-α and IFN-γ and diagnosis of lung cancer. Besides, we observed a synergistic effect of these five pyroptosis-related cytokines in diagnosing lung cancer patient, suggesting their potential as biomarkers for lung cancer diagnosis. Moreover, the combination of TNF-α, MIP-1α and MIP-1β are also potential predictors for the early diagnosis of lung cancer.

## 1 Introduction

Lung cancer including non-small cell lung cancer (NSCLC) and small-cell lung cancer (SCLC) is a global health concern and remains the leading cause of cancer-related mortality [1]. In recent years, advances in diagnostic methods, including computed tomography (CT) and various plasma tumor biomarkers, including carcinoembryonic antigen (CEA), squamous cell carcinoma antigen (SCCA), Cytokeratin 19 fragment (CYFRA21-1) and Neurospecific enolase (NSE), have greatly contributed to the diagnosis and progression assessment of lung carcinoma [2]. Due to the improvement of diagnostic method and standard treatment, the mortality of lung cancer is gradually decreased in recent years [3,4]. However, many lung cancer patients are still being diagnosed at an advanced stage, leading to a poor prognosis. The overall 5-year survival rate for lung cancer patients is still only 19.7 % in China and 24 % in the United States [4,5]. One factor that contribute this result is that many patients are fear of radiation damage from X-rays or CT examinations, which lead to many patients decide to get an X-ray or CT scan finally until they experience discomfort and decide to seeking medical attention. In addition, the examination of traditional lung cancer biomarkers is typically conducted after the lung lesions are already visible. Hence, it is imperative to develop a more convenient and acceptable diagnostic method for lung cancer patients.

In our previous studies, we demonstrated that pyroptosis-related cytokines TNF-α, IFN-γ, IP-10, MIP-1α, MIP-1β and MIP-2 can induce T cell infiltration into lung cancer tissues and ultimately promote the lung cancer tissue regression [6]. While normal lung cells switch to lung cancer cells, the cancerous cells were immediately identified by immune cells [7]. TNF-α, IFN-γ, IP-10, MIP-1α, MIP-1β and MIP-2 as indispensable immune factors is necessary in regulating anti-cancer immune. IP-10, MIP-1α, MIP-1β and MIP-2 as chemokines induce T cell infiltration into tumor tissue [8,9]. Infiltrated T cells releases TNF-α and IFN-γ to kill cancer cells [10,11]. However, evidence suggests that these anti-tumor factors are unable to rapidly eliminate tumor cells within the body, which results in the immune system need to continuously release these factors. Hence, the abnormal concentration of these factors maybe means the occurrence of lung carcinoma. In addition, elevated levels of TNF-α or IFN-γ is linked with the development of lung cancer. Increased MIP-1α, MIP-1β or MIP-2 expression were also associated with poorer prognosis and decreased survival rate in lung adenocarcinoma patients. Nonetheless, whether the combination examination of these factors is benefitted to found out lung cancer is still needed to be clarified.

Furthermore, the detection of these factors can be easily completed using patient blood samples, which provide a convenient, inexpensive, and non-invasive approach. Therefore, in this study, we detect the concentrations of TNF-α, IFN-γ, IP-10, MIP-1α, MIP-1β and MIP-2 in collected blood samples from both lung cancer patients and healthy volunteers, to elucidate the potential association between these pyroptosis-related cytokines and lung carcinoma.

## 2 Materials and methods

### 2.1 Patient samples

Plasma samples were collected from 258 primary lung cancer patients and 80 healthy volunteers at the Third Xiangya Hospital of Central South University (Changsha, China) from May 2021 to July 2023. The study was approved by the Ethics Committees of the Third Xiangya Hospital, Central South University (No. 23135). The blood samples used in this study is the remaining part from previous studies and frozen in the Biobank of Third Xiangya Hospital. Inclusion criteria: All Patients in the study were new diagnosed lung cancer, and diagnosed by pathology. In addition, none of the lung cancer patients received chemotherapy, radiotherapy, or surgery before treatment and blood sampling. Exclusion criteria: Patients who had undergone anticancer treatment, such as radiotherapy and chemotherapy; patients with severe infection; patients with severe hepatic and renal dysfunction; patients with other malignant tumors; patients with acute myocardial infarction, unstable angina, uncontrollable hypertension, and symptomatic sustained arrhythmia within 6 months; patients with communication and cognitive impairment. Detailed clinical characteristics of patients and healthy volunteers were shown in Table 1.

**Table 1:** Characteristics and clinical data of lung cancer patients and healthy volunteers in the study.

Characteristics	Lung cancer (n = 258) n (%)	Healthy (n = 80) n (%)	*P*
Age, y Mean (SD)	61.5 (10.81)	60.9 (7.68)	0.687
Gender			0.090
Female	92 (36.00)	30 (37.00)	
Male	166 (64.00)	50 (63.00)	
BMI (kg/m^2^)	23.3 (3.20)	24.0 (2.34)	0.054
Smoking status			0.153
No	128 (50.00)	42 (52.50)	
Yes	128 (50.00)	38 (47.50)	
Alcohol drinking			0.110
No	193 (74.81)	59 (73.75)	
Yes	63 (25.19)	21 (26.25)	
Cancer pathology			
Squamous	69 (26.74)	–	
Adenocarcinoma	159 (61.63)	–	
Other	30 (11.63)	–	
TNM stage			
0	6 (2.33 %)	–	
I	42 (16.28 %)	–	
II	57 (22.09 %)	–	
III	69 (26.74 %)	–	
IV	84 (32.56 %)	–	
IFN-γ, pg/ml, median (interquartile range)	5.9 (3.5–9.7)	12.38 (9.1–27.8)	＜0.0001*
IP-10, pg/ml, median (interquartile range)	57.2 (43.0–76.5)	45.5 (37.1–56.7)	＜0.0001*
MIP-1α, pg/ml, median (interquartile range)	115.4 (96.6–191.2)	34.4 (21.8–75.2)	0.0004*
MIP-1β, pg/ml, median (interquartile range)	160.5 (124.9–218.6)	49.3 (25.6–78.7)	＜0.0001*
MIP-2, pg/ml, median (interquartile range)	77.9 (50.1–186.5)	22.6 (17.8–31.2)	＜0.0001*
TNF-α, pg/ml, median (interquartile range)	10.3 (5.7–16.6)	3.80 (2.3–6.2)	＜0.0001*
CEA, pg/ml, median (interquartile range)	4.5 (2.6–16.2)	2.1 (1.3–3.4)	0.0154*

Notes: Normally distributed data were expressed as mean ± standard deviation (SD), and the skewed data were presented as median (interquartile range). *P＜0.05.

BMI = body mass index; - = Not applicable.

### 2.2 Cytometric Bead Array

The levels of IFN-γ, TNF-α, MIP-1α, MIP-1β, MIP-2, and IP-10 levels from patient plasmas were measured using a Multiplex Luminex assay (BD sciences). Reagents for quantitative ProcartaPlex Luminex immunoassay were sourced from Affymetrix eBioscience. Cytometric Bead Array (R&D) were performed according to the manufacturer’s instructions. and results were read on the Bio-Plex 200 instrument.

### 2.3 Quantification and statistical analysis

Statistical analysis was performed using SPSS version 18.0 (SPSS; IBM Inc., Chicago, IL, USA) and GraphPad Prism 9 (GraphPad Software, San Diego, CA, USA). The normally distributed data were expressed as mean ± standard deviation (SD), and the skewed data were presented as median (interquartile range). Independent-sample-t test was used for comparison of variable between two groups. One-way analysis of variance was used for comparison of multiple groups. Count data were compared using the χ2 test. The receiver operator characteristic curve (ROC) was used to evaluate the diagnostic efficacy of biomarkers for lung cancer. The area under the ROC curve (AUC) was used to evaluate the predictive accuracy of biomarkers in the lung cancer diagnosis. Pearson correlation coefficient analysis method was used for correlation analysis. All P-values were two-sided and P < 0.05 was considered statistically significant.

## 3 Results

### 3.1 Baseline characteristics and clinical data of the two groups

Characteristics and clinical data of lung cancer patients and healthy volunteers in the study are shown in Table 1. The study included in 258 primary lung cancer patients and 80 control subjects. The mean age of them was 61.5 ± 10.81 and 60.9 ± 7.68. Baseline of characteristics of these individuals, including gender, age, body mass index (BMI), smoking status and alcohol drinking have no statistically difference between the two groups. Compared to healthy volunteers, the levels of IP-10, MIP-1α, MIP-1β, MIP-2, and TNF-α were significantly higher in lung cancer patients (45.5 (37.1–56.7): 57.2 (43.0–76.5), 34.4 (21.8–75.2): 115.4 (96.6–191.2), 49.3 (25.6–78.7): 160.5 (124.9–218.6), 22.6 (17.8–31.2): 77.9 (50.1–186.5), 3.80 (2.3–6.2): 10.3 (5.7–16.6)), but the level of IFN-γ was decreased in lung cancer patients (12.38 (9.1–27.8): 5.9 (3.5–9.7)). Since smoking and alcohol drinking may affect the level of pyroptosis-related cytokines in lung cancer patients, we further divided lung patients into smoking and non-smoking groups, or drinking and non-drinking groups, and then compared the levels of TNF-α, IFN-γ, IP-10, MIP-1α, MIP-1β and MIP-2 in different group. Results showed that the expression levels of these factors are slight difference between smoking and non-smoking group, or drinking and non-drinking group (Table 2), suggesting that smoking or drinking are minor factors to influence the expression of pyroptosis-related cytokines.

**Table 2:** The level of cytokines in lung cancer patients with different habit.

Characteristics	IFN-γ, pg/ml, median (interquartile range)	IP-10, pg/ml, median (interquartile range)	MIP-1α, pg/ml, median (interquartile range)	MIP-1β, pg/ml, median (interquartile range)	MIP-2, pg/ml, median (interquartile range)	TNF-α, pg/ml, median (interquartile range)
Smoking status						
No	6.2 (3.9–9.7)	54.9 (42.6–71.8)	106.5 (95.2–178.4)	155.3 (121.9–199.0)	72.2 (44.0–160.5)	11.3 (5.8–19.0)
Yes	5.7 (3.4–9.8)	58.4 (42.4–79.5)	164.1 (100.2–202.3)	183.9 (129.7–222.7)	101.2 (51.1–211.0)	10.0 (5.7–16.5)
*P*	0.6048	0.1911	<0.0001	0.1385	0.0244	0.3289
Alcohol drinking						
No	6.2 (3.7–10.0)	56.5 (42.3–77.5)	114.4 (96.4–189.7)	158.7 (124.9–204.9)	73.7 (45.3–165.2)	9.9 (5.6–17.0)
Yes	5.4 (2.7–9.3)	62.6 (43.3–75.0)	162.9 (101.8–202.3)	175.5 (124.9–224.2)	126 (57.9–292.7)	11.4 (6.4–16.4)
*P*	0.4367	0.3971	0.0583	0.0835	0.0030	0.8915

### 3.2 The diagnostic value of biomarkers in the lung cancer

**Fig. 1:**
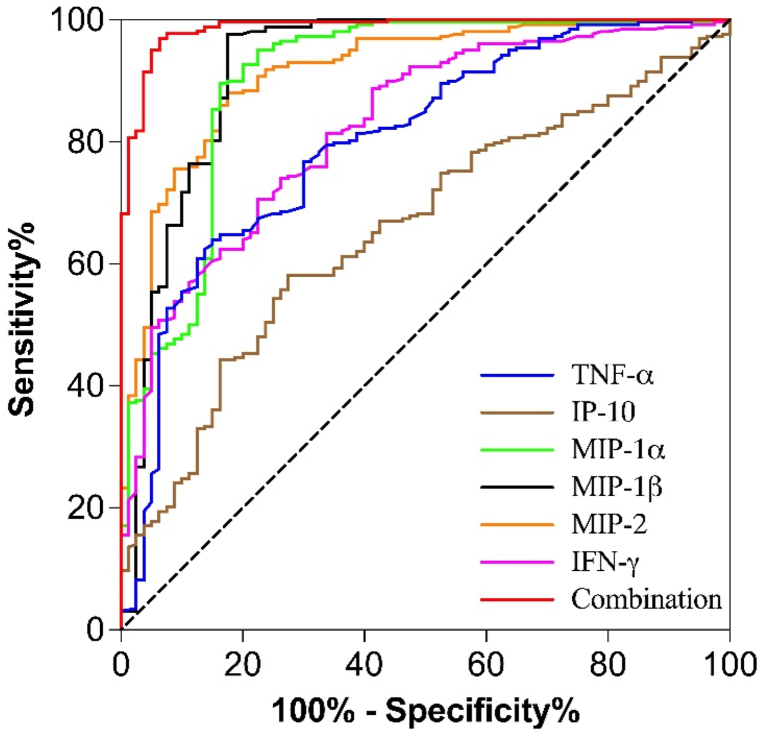
ROC curve analysis of IFN-γ, IP-10, MIP-1α, MIP-1β, MIP-2, TNF-α and biomarkers combination (not included in IP-10) in the diagnosis of lung cancer.

**Fig. 2:**
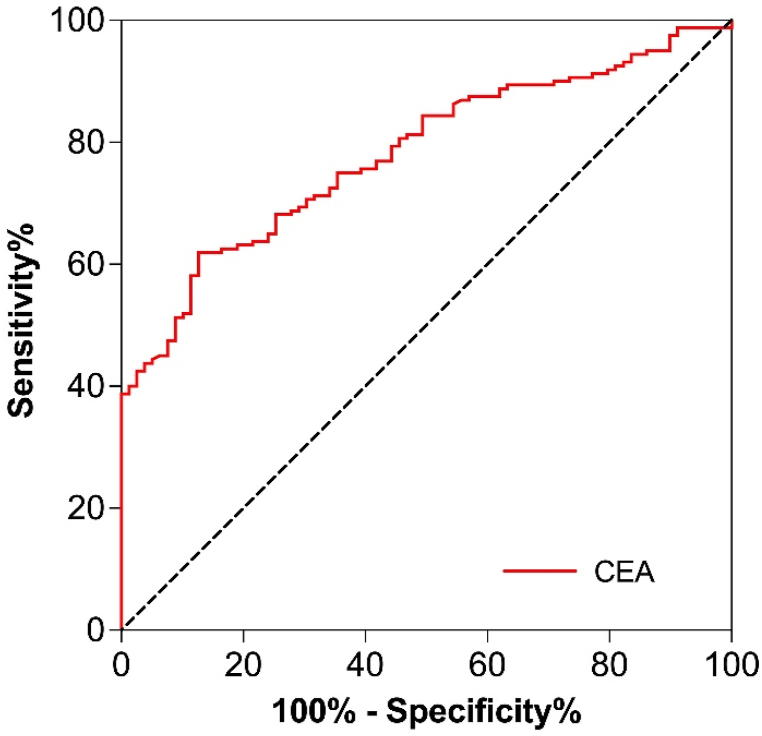
ROC curve analysis of CEA in the diagnosis of lung cancer.

Table 3 showed the cut-off levels, the area under the ROC curve (AUC) values, sensitivity, specificity, and Youden index of the inflammatory cytokines in lung cancer diagnosis. ROC curve analysis showed that IFN-γ, IP-10, MIP-1α, MIP-1β, MIP-2, and TNF-α had a significantly predictive efficiency in the diagnosis of lung cancer, and the AUC values of the above biomarkers were 0.800, 0.656, 0.905, 0.921, 0.914, and 0.824 (Table 3 and Fig. 1). To investigate whether the combination of above cytokine provides stronger connection to the diagnosis of cancer than each individual cytokine alone, we also evaluated the relevance between combined inflammatory cytokine score and cancer. Results showed that the AUC increased to 0.986 when the above factors were combined. In addition, the diagnostic value of these biomarkers in the diagnosis of lung cancer is reflected by their high sensitivity and specificity. IFN-γ, IP-10, MIP-1α, MIP-1β, MIP-2 and TNF-α had a sensitivity of 64.0 %, 58.1 %, 89.9 %, 97.7 %, 88.0 % and 70.5 %, and specificity of 85.0 %, 72.5 %, 83.8 %, 82.5 %, 82.5 % and 77.5 %, respectively. and the combination of these factors exhibits a higher diagnostic efficiency (AUC = 0.986) than individual biomarkers, with sensitivity and specificity of 96.9 % and 93.7 %, respectively (Table 3). Due to CEA is an important indicator for the currently clinical diagnose of lung cancer, we also detected the correlation between CEA and the occurrence of lung cancer in the same samples. In our results, although the level of CEA is increased in part of lung cancer patients, the AUC values of CEA is only 0.778 (Table 1, Table 3 and Fig. 2), which is markedly lower than the combination of pyroptosis-related cytokines. Therefore, these results suggest that the combination detection of IFN-γ, IP-10, MIP-1α, MIP-1β, MIP-2 and TNF-α maybe provide better accuracy, sensitivity and specificity in the diagnosis of lung cancer.

**Table 3:** The diagnostic efficacy of biomarkers in lung cancer patients and healthy volunteers.

Variables	AUC	95 % CI	*P*	Youden index	Cut-off value	Sensitivity	Specificity	Positive LR	Negative LR
CEA	0.778	0.720–0.835	<0.0001	0.487	3.87	61.4	87.3	4.83	0.44
IFN-γ	0.800	0.744–0.857	<0.0001	0.490	8.38	64.0	85.0	4.27	0.42
IP-10	0.656	0.592–0.721	<0.0001	0.306	53.05	58.1	72.5	1.73	0.47
MIP-1α	0.905	0.860–0.950	<0.0001	0.737	56.42	89.9	83.8	5.55	0.12
MIP-1β	0.921	0.877–0.966	<0.0001	0.802	88.69	97.7	82.5	5.58	0.03
MIP-2	0.914	0.879–0.950	<0.0001	0.705	34.59	88.0	82.5	5.03	0.15
TNF-α	0.824	0.773–0.874	<0.0001	0.480	3.80	70.5	77.5	2.63	0.32
Combination	0.986	0.975–0.998	<0.0001	0.906	–	96.9	93.7	15.38	0.03

Notes: Normally distributed data were expressed as mean ± standard deviation (SD), and the skewed data were presented as median (interquartile range). *P＜0.05.

- = Not applicable.

### 3.3 Correlation between the biomarkers and clinical stage of lung cancer

To further explore the correlation between the biomarkers’ level and occurrence of lung cancer (including TNM 0, I, II, III, IV stage), we performed Pearson correlation analysis. The results indicated that TNF-α (r = 0.400, P < 0.01), MIP-2 (r = 0.343, P < 0.01), MIP-1α (r = 0.551, P < 0.01) and MIP-1β (r = 0.403, p < 0.01) were positively association with the occurrence of lung cancer, but IFN-γ (r = −0.483, p < 0.01) was negatively association with the occurrence of lung cancer (Table 4). These results indicated that the above three cytokines were associated with progression of lung cancer.

**Table 4:** The correlation between the biomarkers and occurrence of lung cancer.

Variable	Lung cancer stage	
	r	*P*
TNF-a	0.400**	<0.01
MIP-2	0.343**	<0.01
IFN-γ	−0.483**	<0.01
MIP-1α	0.551**	<0.01
MIP-1β	0.403**	<0.01
IP-10	0.210**	<0.01

### 3.4 The early diagnostic value of biomarkers in the lung cancer

**Fig. 3:**
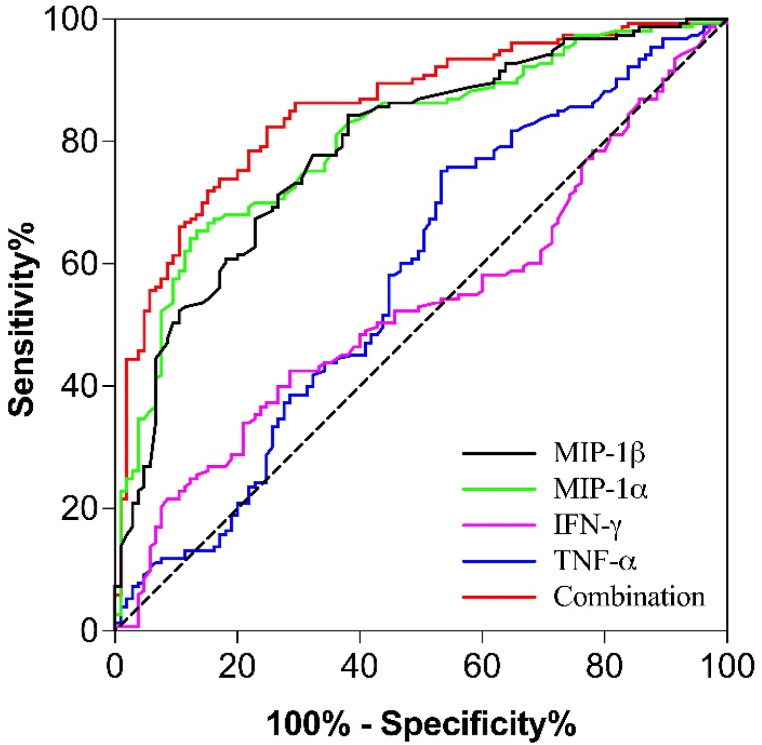
ROC curve analysis of IFN-γ, TNF-α, MIP-1α, MIP-1β and biomarkers combination in early and advanced stage of lung cancer patients.

The early diagnosis of lung cancer is an important clinical problem. To further evaluate the early diagnosis value of the above six biomarkers in lung cancer, we divided the lung cancer patients into two groups of early stage (0, I and II) and late-stage (III and IV) patients and then performed the ROC curve analysis (Table 5 and Fig. 3). The results showed that the AUC values of IFN-γ was 0.533 (P = 0.374), while TNF-α, MIP-1α and MIP-1β were 0.577, 0.804 and 0.791 (P < 0.05), respectively. while combined these three factors, the AUC value was up to 0.854 (P < 0.0001), suggesting high diagnostic accuracy. Besides, the sensitivity of TNF-α, MIP-1α, MIP-1β and combination were 75.2 %, 65.4 %, 84.3 % and 82.4 %, with the specificity was 46.7 %, 86.7 %, 61.9 % and 57.6 %. These results suggested that the combination of MIP-1α, MIP-1β and TNF-α also can effectively predict the occurrence of lung cancer in early stage.

**Table 5:** The early diagnostic efficacy of biomarkers in lung cancer patients.

Variables	AUC	95 % CI	*P*	Youden index	Cut-off value	Sensitivity	Specificity	Positive LR	Negative LR
IFN-γ	0.533	0.462–0.603	0.374	0.139	8.21	42.5	71.4	0.673	1.242
TNF-α	0.577	0.505–0.650	0.035	0.219	6.95	75.2	46.7	0.709	1.883
MIP-1α	0.804	0.750–0.857	<0.0001	0.521	162.7	65.4	86.7	0.203	2.506
MIP-1β	0.791	0.735–0.846	<0.0001	0.462	146.15	84.3	61.9	0.452	3.943
Combination	0.854	0.653–0.870	<0.0001	0.576	–	82.4	57.6	0.301	4.273

## 4 Discussion

In this study, we found that pyroptosis-related cytokines IFN-γ, IP-10, MIP-1α, MIP-1β, MIP-2 and TNF-α are the potential markers for lung cancer diagnosis. The combination of these cytokines showed better accuracy, sensitivity and specificity in lung cancer diagnose. Pearson correlation analysis also revealed significant associations between these cytokines and the occurrence of lung cancer. Additionally, the ROC curve indicated that combination of MIP-1α, MIP-1β, and TNF-α is a good predictor to the early stage of lung cancer.

In our previous studies, we found that the adaptive immunity which against lung carcinoma can be activated by pyroptotic lung cancer cells, which lead to significant increase of IFN-γ, IP-10, MIP-1α, MIP-1β, MIP-2, and TNF-α [6]. However, this phenotype cannot fully explain the abnormal elevation of chemokines and cytokines in all stages of lung cancer patients. Besides, lung cancer cells and immune cells are known to produce MIP-1α, MIP-1β and MIP-2 [12,13]. When lung tissue cells transform into lung cancer cells, immune cells release these chemokines to facilitate infiltration into tumor tissue and release TNF-α and IFN-γ to eliminate the cancer cells [14]. Since immune cells continuously recognize and eliminate tumor cells, this process may play a pivotal role in maintaining the sustained high levels of TNF-α, IFN-γ, MIP-1α, MIP-1β, and MIP-2 in the plasma.

Furthermore, these chemokines and cytokines have been long implicated in the development and progression of cancer. For instance, TNF-α, despite its anti-tumor properties, has long been implicated in the development of various cancers, including lung cancer, gastric cancer, pancreatic cancer, and liver cancer [[15], [16], [17], [18]]. Similar with our results, elevated levels of TNF-α have been considered to be linked to an increased risk of lung cancer. IFN-γ, secreted from T cells and natural killer cells, exhibits an anti-tumor role. However, when secreted by tumor-associated macrophages, it can promote the growth of cancer cells [19,20]. Previous studies indicate that high levels of IFN-γ were associated with poorer prognosis and worse clinical outcomes in lung cancer patients [21]. In our study, we found that TNF-α alone as a diagnostic biomarker for lung carcinoma lacks sufficient sensitivity (70.5 %) and specificity (77.5 %). IFN-γ presented a better specificity (85.0 %) but poor sensitivity (64.0 %) for lung cancer diagnosis. Nevertheless, both cytokines were correlated with the occurrence of lung cancer. TNF-α was positively associated with lung cancer, while IFN-γ exhibits negative association. However, the two factors may not be enough to determine the occurrence of early cancer accurately. Combining them with other indicators might provide a more reliable diagnosis.

MIP-1α, MIP-1β and MIP-2, as important chemokines, play pivotal role in attracting immune cells to tumor tissues sites [22,23]. However, other studies also found that higher expression of MIP-1α, MIP-1β or MIP-2 were also associated with poorer prognosis and decreased survival rate in lung adenocarcinoma patients [[24], [25], [26]]. High levels of MIP-1α have also been linked to promoting cancer cells growth and survival by stimulating tumor neovascularization [27,28]. Increased MIP-1β not only contributes to the tumor angiogenesis and therapeutic response during radiotherapy, but also takes part in the metastasis of lung cancer [[29], [30], [31]]. Although MIP-2 has not been extensively studied in relation to lung carcinoma, higher expression of MIP-2 has been confirmed to be associated with higher lung cancer stages [32]. In our study, we found that MIP-1α, MIP-1β and MIP-2 exhibit a better sensitivity (89.9 %, 97.7 % and 88.0 %) and specificity (83.8 %, 82.5 % and 82.5 %) for lung cancer diagnosis. However, when combined with TNF-α and IFN-γ, the combination of these five pyroptosis-related cytokines exhibited higher sensitivity (96.9 %) and specificity (93.7 %) for lung cancer compared to individual markers.

However, there are still some limitations in this study. Firstly, it is essential to expand the sample size to further validate the value of this test. Additionally, the specific increase of pyroptosis-related cytokines may not be exclusive to lung cancer, the diagnostic role of pyroptosis-related cytokines in other type of tumor still need to further study. Moreover, developed a formula in the future is better to predict the association between the level of pyroptosis-related factors and lung cancer occurrence. However, our findings hold potential clinical and translational implications. Firstly, this study demonstrated that a combination of five factors provides excellent accuracy for the occurrence of lung cancer, which is higher than traditional lung cancer indicator CEA. Secondly, the levels of pyroptosis-related cytokines can be conveniently, inexpensively, and non-invasively measured by CBA or ELISA assay in patient’s blood samples. This makes it suitable for rapid lung cancer diagnosis. Integrating the examination of these pyroptosis-related factors into routine blood tests is likely to be well-received by most patients. Additionally, these markers could help to identify patients who may benefit from pro-pyroptosis treatment or T cell immunity treatment. Furthermore, this detection may provide an earlier diagnose for lung cancer. Overall, this examination holds significant promise for enhancing the detection of lung carcinoma.

## Data availability statement

The data that supports the findings of this study are available in the supplementary material of this article.

## Ethics declarations

This study was reviewed and approved by the Ethics Committee of the Third Xiangya Hospital, Central South University (No. 23135).

## CRediT authorship contribution statement

**Zhouyangfan Peng:** Investigation, Formal analysis, Data curation. **Xiqing Tan:** Resources, Investigation, Data curation. **Yang Xi:** Validation, Formal analysis. **Zi Chen:** Supervision, Data curation. **Yapei Li:** Writing – review & editing, Writing – original draft, Data curation, Conceptualization.

## Declaration of competing interest

The authors declare that they have no known competing financial interests or personal relationships that could have appeared to influence the work reported in this paper.
