# Supplementary material for: Role of pyroptosis-related cytokines in the prediction of lung cancer
Source: Heliyon. 2024 May 15;10(10):e31399. doi: 10.1016/j.heliyon.2024.e31399 (PMC11133917; doi:10.1016/j.heliyon.2024.e31399)
Supplement: Multimedia component 1 [file mmc1.docx]

**Supplemental table1** Detail clinical data of lung cancer patients and control participants

| **ID** | **Pathology** | **Gender** | **Age (Years)** | **Smoking status** | **Alcohol drinking** | **T** | **N** | **M** | **Clinical**  **grading** | **TNF-α**  **(pg/ml)** | **IP-10 (pg/ml)** | **MIP-2**  **(pg/ml)** | **IFN-γ**  **(pg/ml)** | **MIP-1α**  **(pg/ml)** | **MIP-1β**  **(pg/ml)** | **CEA**  **（ng/ml）** |
| --- | --- | --- | --- | --- | --- | --- | --- | --- | --- | --- | --- | --- | --- | --- | --- | --- |
| Patient-1 | LUAD | Female | 65 | No | No | 4 | 3 | 1 | IV | 11.28 | 113.41 | 71.9 | 4.32 | 217.5 | 184.4 | NA |
| Patient-2 | LUAD | Male | 77 | Yes | Yes | 4 | 3 | 1 | IV | 12.72 | 75.00 | 489.5 | 5.14 | 162.9 | 147.9 | NA |
| Patient-3 | LUAD | Male | 65 | Yes | Yes | 4 | 0 | 1c | IVB | 5.79 | 56.62 | 167.5 | 3.45 | 78.8 | 148.1 | NA |
| Patient-4 | LUAD | Male | 64 | Yes | No | 4 | 3 | 1 | IV | 3.89 | 73.01 | 96.1 | 4.21 | 176.7 | 108.3 | 23.24 |
| Patient-5 | LUAD | Male | 74 | Yes | No | 2b | 2 | 1c | IVB | 17.65 | 272.84 | 188.2 | 20.07 | 306.7 | 250.0 | 18.51 |
| Patient-6 | LUAD | Male | 62 | No | No | 4 | 3 | 1a | IVA | 7.79 | 45.29 | 43.7 | 8.34 | 89.7 | 108.8 | 28.95 |
| Patient-7 | LUSC | Male | 64 | No | No | 3 | 3 | 1c | IVB | 8.66 | 60.46 | 194.7 | 3.39 | 168.5 | 107.7 | 30.46 |
| Patient-8 | LUAD | Female | 77 | No | No | 3 | 2 | 0 | IIIB | 6.49 | 108.73 | 244.9 | 5.98 | 165.6 | 149.5 | NA |
| Patient-9 | LUSC | Male | 72 | Yes | No | 4 | 3 | 1a | IVA | 9.18 | 49.92 | 84.5 | 6.37 | 96.5 | 118.3 | 1.12 |
| Patient-10 | LUSC | Male | 56 | No | No | 4 | 0 | 0 | IIIA | 13.41 | 194.32 | 218.5 | 17.13 | 264.7 | 218.2 | 6.29 |
| Patient-11 | LUAD | Female | 79 | No | No | 3 | 2 | 1a | IVA | 7.12 | 110.10 | 96.4 | 12.33 | 178.5 | 147.7 | 4.65 |
| Patient-12 | SCLC | Male | 79 | Yes | Yes | 2b | 2 | 0 | IIIA | 7.08 | 119.50 | 57.9 | 3.48 | 218.7 | 147.6 | 4.52 |
| Patient-13 | LUSC | Male | 69 | Yes | Yes | 3 | 3 | 1 | IV | 7.14 | 92.47 | 211.3 | 4.58 | 163.4 | 107.6 | NA |
| Patient-14 | LUAD | Male | 68 | Yes | No | 4 | 1 | 0 | IIIA | 18.36 | 198.05 | 106.9 | 5.94 | 267.7 | 186.8 | 1.46 |
| Patient-15 | LUSC | Male | 71 | Yes | Yes | 2 | 1 | 0 | IIB | 6.48 | 42.07 | 135.5 | 4.59 | 96.4 | 104.6 | NA |
| Patient-16 | LUSC | Male | 52 | No | No | 2b | 2 | 0 | IIIA | 4.43 | 45.38 | 188.9 | 3.59 | 95.5 | 149.9 | NA |
| Patient-17 | LUSC | Male | 61 | Yes | No | 3 | 3 | 1a | IVA | 5.96 | 48.25 | 42.3 | 3.52 | 96.2 | 157.6 | 6.56 |
| Patient-18 | LUAD | Female | 63 | No | No | 2a | 0 | 0 | IB | 3.19 | 53.39 | 58.7 | 4.61 | 93.3 | 156.7 | 2.2 |
| Patient-19 | NSCLC | Male | 63 | Yes | No | 4 | 3 | 1 | IV | 7.06 | 58.22 | 144.1 | 42.80 | 91.5 | 158.7 | NA |
| Patient-20 | LUSC | Male | 69 | Yes | No | 3 | 0 | 0 | IIA | 5.49 | 41.79 | 71.9 | 1.39 | 98.5 | 101.4 | 19.08 |
| Patient-21 | LUAD | Male | 55 | Yes | Yes | 2a | 2 | 1a | IVA | 7.22 | 97.16 | 33.2 | 2.49 | 163.0 | 102.4 | 28.83 |
| Patient-22 | LUAD | Male | 68 | Yes | Yes | 1c | 1 | 0 | IIB | 4.49 | 37.21 | 32.7 | 6.58 | 93.5 | 110.5 | 5.6 |
| Patient-23 | LUSC | Male | 61 | Yes | Yes | 4 | 3 | 0 | IIIB | 13.49 | 55.53 | 46.8 | 4.38 | 164.6 | 149.5 | 4.12 |
| Patient-24 | SCLC | Male | 67 | Yes | No | 1 | 0 | 1c | IVB | 7.01 | 87.43 | 96.1 | 1.31 | 163.5 | 113.6 | 33.57 |
| Patient-25 | LUSC | Male | 48 | Yes | Yes | 4 | 3 | 0 | IIIC | 4.98 | 51.06 | 458.8 | 9.11 | 91.4 | 120.6 | 1.99 |
| Patient-26 | LUAD | Male | 63 | No | Yes | 2a | 1 | 1b | IVA | 5.51 | 41.71 | 414.9 | 2.39 | 92.5 | 159.9 | 22.38 |
| Patient-27 | LUAD | Male | 64 | Yes | No | 2a | 3 | 1c | IVB | 10.58 | 89.00 | 294.0 | 3.93 | 169.8 | 149.6 | 3.67 |
| Patient-28 | LUAD | Male | 64 | Yes | Yes | 3 | 0 | 0 | IIB | 6.79 | 41.42 | 357.7 | 7.59 | 95.6 | 175.5 | 2.3 |
| Patient-29 | LUSC | Male | 61 | Yes | No | 4 | 2 | 1c | IVB | 8.49 | 156.33 | 529.4 | 14.31 | 219.6 | 157.5 | 4.42 |
| Patient-30 | SCLC | Male | 50 | Yes | No | 4 | 3 | 1c | IVB | 7.78 | 93.58 | 259.4 | 4.49 | 163.5 | 163.6 | 0.74 |
| Patient-31 | LUAD | Male | 54 | Yes | Yes | 4 | 2 | 1c | IVB | 11.79 | 56.69 | 432.7 | 5.43 | 90.6 | 167.4 | 2.73 |
| Patient-32 | LUAD | Female | 59 | No | No | 2b | 3 | 0 | IIIB | 10.66 | 133.87 | 72.4 | 10.21 | 218.5 | 164.6 | NA |
| Patient-33 | LUAD | Female | 41 | No | No | 1b | 0 | 0 | IA3 | 4.53 | 64.06 | 135.5 | 5.31 | 95.7 | 109.6 | 0.06 |
| Patient-34 | LUAD | Male | 52 | Yes | No | x | x | 1c | IVB | 8.89 | 60.21 | 810.4 | 1.23 | 94.7 | 185.7 | 109.2 |
| Patient-35 | LUSC | Female | 57 | No | No | 2a | 0 | 0 | IB | 5.69 | 64.54 | 277.3 | 3.40 | 168.6 | 169.4 | 1.21 |
| Patient-36 | LUAD | Male | 65 | Yes | No | 3 | 2 | 0 | IIIB | 3.83 | 56.54 | 71.7 | 4.39 | 94.6 | 100.4 | 13.93 |
| Patient-37 | LUAD | Male | 55 | Yes | No | 1b | 0 | 0 | IA2 | 5.71 | 37.60 | 212.3 | 9.42 | 96.6 | 101.3 | NA |
| Patient-38 | LUAD | Male | 47 | No | Yes | 1c | 0 | 0 | IA2 | 5.09 | 59.71 | 212.8 | 6.76 | 96.1 | 98.3 | NA |
| Patient-39 | LUSC | Male | 56 | No | No | 1c | x | 0 | IA3 | 3.11 | 36.63 | 229.5 | 5.44 | 96.4 | 150.3 | 3.25 |
| Patient-40 | LUAD | Female | 55 | No | No | 1b | 0 | 0 | IA2 | 7.18 | 88.78 | 38.8 | 4.52 | 166.6 | 108.3 | 1.36 |
| Patient-41 | LUAD | Male | 65 | Yes | No | 1b | 0 | 0 | IA2 | 5.08 | 56.79 | 71.6 | 5.38 | 162.4 | 104.3 | 0.67 |
| Patient-42 | LUSC | Male | 71 | Yes | Yes | 3 | 0 | 0 | IIB | 6.42 | 62.59 | 234.8 | 9.59 | 162.5 | 153.5 | 2.46 |
| Patient-43 | SCLC | Male | 68 | Yes | Yes | 3 | 3 | 1b | IVA | 5.89 | 47.09 | 268.5 | 2.19 | 92.5 | 158.7 | NA |
| Patient-44 | LUSC | Male | 80 | Yes | Yes | x | 3 | x | III | 10.94 | 56.37 | 228.7 | 4.34 | 161.9 | 103.4 | 26.19 |
| Patient-45 | LUSC | Male | 58 | Yes | No | 3 | 0 | x | II | 6.76 | 57.13 | 417.5 | 5.45 | 96.9 | 159.5 | 2.03 |
| Patient-46 | LUAD | Male | 72 | Yes | Yes | 3 | 2 | 0 | IIIB | 7.02 | 55.36 | 743.6 | 6.49 | 96.6 | 183.5 | 20.63 |
| Patient-47 | LUAD | Male | 34 | Yes | Yes | Tis | 0 | 0 | 0 | 3.29 | 53.99 | 88.6 | 10.68 | 97.0 | 100.5 | 0.74 |
| Patient-48 | LUSC | Male | 64 | No | No | 2a | 2 | 1c | IVB | 5.59 | 36.92 | 117.0 | 9.32 | 165.7 | 1286.0 | 3.08 |
| Patient-49 | LUAD | Male | 69 | Yes | Yes | 1b | 0 | 0 | IA2 | 6.78 | 41.78 | 189.8 | 8.64 | 96.5 | 106.6 | NA |
| Patient-50 | LUAD | Female | 53 | No | No | 3 | 2 | 1a | IV | 7.10 | 34.65 | 145.7 | 9.53 | 93.5 | 104.5 | 64.79 |
| Patient-51 | LUAD | Female | 64 | No | No | 1b | 0 | 0 | IA2 | 5.22 | 39.53 | 41.0 | 5.48 | 90.6 | 103.3 | NA |
| Patient-52 | LUAD | Female | 54 | No | No | Tis | 0 | 0 | 0 | 1.48 | 45.83 | 219.8 | 9.30 | 86.4 | 148.5 | 1.31 |
| Patient-53 | LUAD | Female | 51 | No | No | 2b | 0 | 0 | IIA | 3.26 | 53.82 | 485.2 | 3.26 | 78.6 | 101.4 | 7.77 |
| Patient-54 | LUAD | Female | 62 | No | No | 1c | 0 | 0 | IA3 | 12.76 | 168.39 | 584.6 | 23.11 | 220.4 | 250.4 | 0.67 |
| Patient-55 | LUAD | Male | 66 | No | Yes | 1b | 0 | 0 | IA2 | 8.97 | 105.19 | 86.6 | 2.17 | 164.5 | 107.7 | NA |
| Patient-56 | SCLC | Male | 61 | Yes | No | 4 | 3 | 1c | IVB | 7.89 | 57.25 | 206.9 | 1.34 | 88.5 | 146.7 | 9.54 |
| Patient-57 | LUSC | Male | 67 | No | No | 1c | 0 | 0 | IA3 | 3.62 | 49.22 | 513.9 | 9.15 | 84.4 | 149.6 | 3.89 |
| Patient-58 | LUAD | Male | 17 | No | No | Tis | 0 | 0 | 0 | 5.48 | 66.80 | 41.8 | 8.68 | 74.5 | 102.3 | NA |
| Patient-59 | LUAD | Male | 59 | Yes | No | 1c | 0 | 0 | IA3 | 6.28 | 80.09 | 42.4 | 32.70 | 78.4 | 104.4 | 4.67 |
| Patient-60 | LUAD | Male | 61 | Yes | Yes | 1c | 0 | 0 | IA3 | 5.55 | 71.32 | 582.9 | 11.51 | 163.3 | 223.5 | 2.94 |
| Patient-61 | LUAD | Male | 72 | Yes | Yes | 2a | 0 | 0 | IB | 19.77 | 65.87 | 612.3 | 26.24 | 307.6 | 308.2 | 4.07 |
| Patient-62 | LUAD | Female | 68 | No | No | 1b | 0 | 0 | IA2 | 6.77 | 38.47 | 135.5 | 9.39 | 56.6 | 105.5 | 0.06 |
| Patient-63 | LUAD | Female | 67 | No | No | 1c | 0 | 1b | IVA | 6.58 | 42.44 | 96.2 | 9.43 | 48.5 | 98.6 | 11.48 |
| Patient-64 | LUAD | Female | 64 | No | No | 1b | 0 | 0 | IA2 | 9.72 | 126.11 | 57.0 | 12.29 | 218.3 | 59.7 | NA |
| Patient-65 | LUSC | Male | 58 | No | No | 2 | 2 | 0 | IIIA | 4.75 | 49.58 | 50.1 | 5.22 | 236.4 | 246.4 | 2.37 |
| Patient-66 | LUAD | Male | 65 | Yes | No | 4 | 3 | 1c | IVB | 6.16 | 38.76 | 210.2 | 2.85 | 177.0 | 252.3 | 4.34 |
| Patient-67 | LUAD | Female | 72 | No | No | 1 | 3 | 1b | IVA | 13.38 | 53.65 | 87.0 | 18.29 | 84.7 | 222.7 | 33.1 |
| Patient-68 | LUSC | Female | 55 | No | No | 3 | 2 | 1 | IV | 9.87 | 45.28 | 24.9 | 15.37 | 187.6 | 222.7 | 0.776 |
| Patient-69 | LUSC | Male | 61 | Yes | No | 2b | 1 | 0 | IIIB | 16.70 | 85.17 | 73.7 | 11.48 | 308.5 | 299.7 | 23.96 |
| Patient-70 | LUSC | Male | 79 | Yes | No | 2b | 1 | 0 | IIIB | 26.90 | 37.78 | 50.1 | 12.45 | 95.1 | 124.9 | 5.15 |
| Patient-71 | LUSC | Male | 60 | Yes | No | 4 | 1 | 0 | IIIA | 15.66 | 45.05 | 31.5 | 8.42 | 180.8 | 121.9 | NA |
| Patient-72 | LUAD | Male | 64 | Yes | No | 1 | 2 | 0 | IIIA | 21.81 | 53.12 | 22.1 | 6.61 | 178.6 | 124.9 | NA |
| Patient-73 | LUAD | Female | 65 | No | No | 4 | 2 | 1c | IVB | 36.50 | 63.38 | 61.7 | 7.03 | 102.5 | 252.3 | 2.09 |
| Patient-74 | NSCLC | Male | 60 | Yes | No | 1c | 2 | 1b | IVA | 43.22 | 69.63 | 300.9 | 1.19 | 281.1 | 261.2 | NA |
| Patient-75 | NSCLC | Female | 55 | No | No | 3 | 2 | 0 | IIIB | 30.71 | 36.61 | 24.7 | 3.97 | 189.7 | 163.4 | 1.06 |
| Patient-76 | LUSC | Male | 61 | No | No | 1c | 2 | 1c | IVB | 28.44 | 52.14 | 522.5 | 3.27 | 237.3 | 225.7 | NA |
| Patient-77 | LUAD | Male | 43 | Yes | No | 3 | 0 | 0 | IIB | 26.16 | 79.74 | 30.8 | 24.00 | 177.0 | 79.0 | NA |
| Patient-78 | LUSC | Male | 56 | Yes | No | 4 | 1 | 0 | IIIA | 20.88 | 153.78 | 39.2 | 14.68 | 282.2 | 184.2 | 2.78 |
| Patient-79 | LUSC | Male | 55 | No | No | 2b | 2 | 0 | IIIA | 18.10 | 161.32 | 51.1 | 1.19 | 103.2 | 114.5 | 5.81 |
| Patient-80 | LUAD | Male | 58 | Yes | No | 2b | 0 | 0 | IIA | 19.54 | 133.80 | 19.8 | 1.05 | 101.2 | 169.3 | NA |
| Patient-81 | LUSC | Male | 66 | Yes | Yes | 3 | 2 | 0 | IIIB | 15.66 | 64.85 | 26.4 | 8.70 | 101.8 | 187.1 | 3.58 |
| Patient-82 | LUAD | Female | 59 | No | No | 1c | 1 | 0 | IIB | 13.62 | 59.46 | 50.1 | 4.80 | 99.1 | 160.5 | NA |
| Patient-83 | LUAD | Female | 58 | No | No | 4 | 3 | 1a | IVA | 8.30 | 41.81 | 185.9 | 5.08 | 97.1 | 222.7 | NA |
| Patient-84 | LUAD | Female | 79 | No | No | 3 | 0 | 0 | IIB | 36.53 | 64.81 | 59.5 | 6.47 | 105.8 | 157.5 | 8.9 |
| Patient-85 | LUAD | Female | 34 | Yes | No | 1 | 1 | 0 | IIA | 2.31 | 43.55 | 39.2 | 5.64 | 177.0 | 83.4 | NA |
| Patient-86 | LUSC | Male | 65 | Yes | No | 2 | 1 | 0 | IIB | 1.74 | 50.33 | 53.1 | 7.03 | 99.8 | 151.6 | 3.33 |
| Patient-87 | LUSC | Male | 74 | No | No | 2a | 0 | 1c | IVb | 8.40 | 39.25 | 343.7 | 9.53 | 178.1 | 188.6 | 3.38 |
| Patient-88 | LUSC | Male | 57 | Yes | No | 3 | 2 | 1c | IVB | 13.45 | 78.31 | 46.2 | 14.68 | 177.5 | 216.8 | 2.87 |
| Patient-89 | SCLC | Male | 70 | No | No | 2a | 2 | 1c | IVB | 3.48 | 42.19 | 475.9 | 15.93 | 237.7 | 240.5 | 738.5 |
| Patient-90 | LUSC | Male | 59 | Yes | No | 1c | 3 | 1a | IVA | 4.39 | 50.11 | 87.0 | 18.43 | 177.0 | 252.3 | 2.82 |
| Patient-91 | LUAD | Male | 74 | Yes | No | 4 | 2 | 1c | IVB | 2.31 | 56.21 | 134.8 | 12.31 | 182.4 | 201.9 | 123.8 |
| Patient-92 | LUAD | Female | 49 | No | No | 3 | 0 | 0 | IIIB | 17.73 | 37.74 | 27.9 | 7.58 | 101.2 | 139.7 | NA |
| Patient-93 | LUSC | Male | 58 | Yes | No | 4 | 1 | 0 | IIIC | 13.58 | 23.04 | 76.3 | 8.70 | 96.4 | 187.1 | NA |
| Patient-94 | LUAD | Female | 37 | No | No | 1b | 1 | 0 | IIB | 9.17 | 22.21 | 34.8 | 10.37 | 98.5 | 222.7 | NA |
| Patient-95 | LUAD | Female | 44 | No | No | 2a | 1 | 0 | IIB | 3.58 | 55.76 | 134.8 | 15.37 | 96.4 | 261.2 | NA |
| Patient-96 | LUAD | Male | 54 | Yes | No | 2 | 3 | 1 | IVB | 6.09 | 29.75 | 190.2 | 13.43 | 236.9 | 234.5 | 52.29 |
| Patient-97 | LUAD | Female | 57 | No | No | 2a | 2 | 0 | IIIA | 8.43 | 36.84 | 89.8 | 13.70 | 101.2 | 133.8 | 8.92 |
| Patient-98 | LUAD | Female | 30 | No | No | 1a | 1 | 0 | IIB | 12.25 | 43.24 | 24.7 | 12.59 | 100.5 | 124.9 | NA |
| Patient-99 | LUSC | Male | 64 | Yes | No | 4 | 2 | 1 | IV | 12.35 | 33.59 | 418.4 | 15.51 | 180.8 | 259.7 | 1.71 |
| Patient-100 | NSCLC | Male | 55 | No | No | 2a | 2 | 0 | IIIA | 18.10 | 43.62 | 73.7 | 19.82 | 100.5 | 154.5 | NA |
| Patient-101 | LUAD | Female | 44 | No | No | 2a | 1 | 0 | IIB | 20.34 | 48.15 | 31.2 | 8.14 | 103.2 | 110.1 | NA |
| Patient-102 | LUAD | Male | 59 | No | No | 1a | 1 | 0 | IIB | 12.85 | 29.07 | 70.6 | 6.75 | 101.8 | 110.1 | NA |
| Patient-103 | LUAD | Female | 60 | No | No | 4 | 3 | 1c | IVB | 22.48 | 33.82 | 214.8 | 9.81 | 176.5 | 222.7 | 22.09 |
| Patient-104 | LUSC | Male | 70 | Yes | No | 2a | 2 | 0 | IVB | 30.04 | 37.66 | 343.7 | 8.70 | 179.7 | 219.7 | NA |
| Patient-105 | LUAD | Female | 53 | No | No | 4 | 1 | 0 | IIIC | 42.42 | 44.15 | 78.9 | 8.42 | 103.2 | 160.5 | 5.92 |
| Patient-106 | SCLC | Male | 57 | No | No | 4 | 2 | 0 | IIIB | 24.16 | 41.74 | 124.3 | 10.92 | 175.9 | 222.7 | 40.56 |
| Patient-107 | LUAD | Male | 67 | Yes | No | 1b | 1 | 0 | IIB | 20.81 | 50.86 | 25.2 | 6.47 | 98.5 | 199.0 | NA |
| Patient-108 | LUAD | Female | 69 | No | No | 3 | 3 | 0 | IIIC | 19.41 | 49.35 | 241.0 | 5.78 | 103.8 | 119.0 | 37.43 |
| Patient-109 | LUSC | Male | 71 | No | No | 2 | 3 | 0 | IIIB | 12.28 | 30.16 | 114.3 | 9.53 | 104.5 | 222.7 | 9.43 |
| Patient-110 | LUAD | Female | 63 | No | No | 3 | 3 | 1c | IVB | 22.08 | 51.69 | 940.3 | 10.37 | 174.8 | 222.7 | NA |
| Patient-111 | LUAD | Male | 71 | Yes | Yes | 2b | 3 | 0 | IIIB | 26.03 | 85.24 | 73.1 | 2.85 | 104.5 | 302.7 |  |
| Patient-112 | LUAD | Female | 67 | No | No | 3 | 3 | 1 | IVB | 31.78 | 51.09 | 183.8 | 11.76 | 179.2 | 1171.0 | 8.91 |
| Patient-113 | LUAD | Male | 58 | Yes | No | 4 | 1 | 0 | IIIA | 4.02 | 37.36 | 155.4 | 0.91 | 178.1 | 157.5 | 3.3 |
| Patient-114 | LUAD | Female | 68 | No | No | 2b | 0 | 0 | IIA | 7.97 | 34.50 | 49.1 | 0.63 | 99.8 | 124.9 | 5.36 |
| Patient-115 | LUAD | Female | 65 | No | Yes | 1 | 0 | 0 | IA | 12.38 | 43.32 | 21.6 | 2.30 | 96.4 | 120.4 | 4.7 |
| Patient-116 | LUAD | Female | 58 | Yes | Yes | 4 | 3 | 1 | IVA | 10.04 | 92.71 | 300.9 | 9.25 | 187.1 | 222.7 | 2.73 |
| Patient-117 | LUAD | Female | 65 | No | No | 1b | 0 | 0 | IA2 | 9.44 | 78.38 | 39.2 | 15.93 | 84.7 | 107.1 | 2.38 |
| Patient-118 | LUAD | Male | 63 | Yes | No | 2a | 0 | 1a | IVA | 9.87 | 222.32 | 441.6 | 12.45 | 238.5 | 296.8 | NA |
| Patient-119 | LUAD | Female | 50 | No | No | 2b | 2 | 0 | IIIA | 16.46 | 36.61 | 168.2 | 13.01 | 236.9 | 204.9 | 21.28 |
| Patient-120 | LUAD | Male | 52 | Yes | No | 1b | 0 | 0 | IA | 20.61 | 76.42 | 91.3 | 13.70 | 94.4 | 130.8 | NA |
| Patient-121 | LUAD | Female | 35 | No | No | 1b | 1 | 0 | IIB | 18.74 | 43.40 | 60.6 | 10.37 | 91.0 | 178.2 | 1.19 |
| Patient-122 | LUAD | Male | 56 | No | No | 1b | 0 | 0 | IA2 | 12.25 | 56.29 | 38.3 | 6.19 | 79.8 | 126.4 | 2.92 |
| Patient-123 | LUAD | Male | 49 | No | No | 1c | 0 | 0 | IA3 | 11.31 | 41.21 | 43.9 | 10.92 | 84.0 | 116.0 | 2.56 |
| Patient-124 | LUAD | Female | 60 | NA | NA | 2a | 1 | 0 | IIB | 11.68 | 64.66 | 70.0 | 5.91 | 92.3 | 282.0 | NA |
| Patient-125 | LUSC | Male | 82 | Yes | No | X | 3 | 1 | IVA | 11.78 | 78.38 | 646.6 | 10.78 | 308.8 | 445.0 | 3.13 |
| Patient-126 | LUAD | Female | 68 | No | No | 1a | 1 | 0 | IIB | 4.95 | 44.98 | 163.2 | 10.09 | 62.6 | 121.9 | NA |
| Patient-127 | LUAD | Female | 60 | No | No | 4 | 3 | 1a | IVA | 5.56 | 50.86 | 43.9 | 3.97 | 53.0 | 101.2 | NA |
| Patient-128 | NSCLC | Female | 49 | No | No | 1b | 1 | 0 | IIB | 6.09 | 35.10 | 61.7 | 4.66 | 93.7 | 62.7 | NA |
| Patient-129 | LUAD | Male | 54 | Yes | No | 1c | 2 | 0 | IIIA | 8.43 | 28.32 | 30.8 | 10.50 | 251.6 | 126.4 | NA |
| Patient-130 | LUSC | Male | 63 | Yes | Yes | 4 | 2 | 0 | IIIB | 17.26 | 36.61 | 74.3 | 18.57 | 190.7 | 193.1 | 1.8 |
| Patient-131 | LUAD | Male | 57 | Yes | No | 4 | 0 | 0 | IIIA | 9.24 | 43.47 | 25.8 | 3.97 | 103.2 | 191.6 | 2.51 |
| Patient-132 | LUSC | Male | 55 | Yes | Yes | 4 | 2 | 1a | IVA | 12.38 | 32.88 | 59.5 | 2.58 | 202.3 | 282.0 | 3.38 |
| Patient-133 | LUAD | Female | 51 | No | No | 2 | X | 1a | IVA | 8.03 | 44.98 | 192.4 | 1.05 | 314.7 | 287.9 | NA |
| Patient-134 | LUAD | Male | 68 | Yes | Yes | 1c | 1 | 0 | IIIA | 10.17 | 22.21 | 50.6 | 1.60 | 194.8 | 129.3 | NA |
| Patient-135 | LUAD | Female | 53 | Yes | Yes | 1c | 1 | 0 | IIIA | 10.78 | 14.93 | 51.1 | 10.09 | 103.2 | 124.9 | 5.45 |
| Patient-136 | LUAD | Male | 68 | Yes | No | 2b | 3 | 0 | IIIC | 12.41 | 10.48 | 75.0 | 3.97 | 111.8 | 157.5 | 12.68 |
| Patient-137 | LUAD | Male | 71 | Yes | No | 4 | 2 | 1 | IV | 15.93 | 18.81 | 94.2 | 0.35 | 192.3 | 173.8 | NA |
| Patient-138 | LUAD | Female | 52 | No | No | 4 | 3 | 1a | IV | 12.25 | 76.87 | 106.3 | 12.31 | 293.9 | 253.8 | 347.7 |
| Patient-139 | LUAD | Male | 59 | Yes | Yes | 2b | 3 | 1b | IVa | 14.12 | 35.10 | 112.7 | 15.37 | 203.7 | 166.4 | 3.95 |
| Patient-140 | LUAD | Female | 75 | No | No | 3 | 3 | 1c | IVb | 15.02 | 10.60 | 61.7 | 18.43 | 252.4 | 213.8 | 39.39 |
| Patient-141 | LUSC | Male | 67 | No | No | 4 | 3 | 0 | IIIC | 14.72 | 14.22 | 52.1 | 9.53 | 191.2 | 124.9 | 25.13 |
| Patient-142 | NSCLC | Male | 70 | Yes | No | 2a | 1 | 1c | IVB | 6.16 | 30.35 | 115.9 | 6.05 | 294.9 | 283.4 | 4.26 |
| Patient-143 | SCLC | Male | 59 | Yes | Yes | 4 | 3 | 0 | IIIC | 15.93 | 14.74 | 51.1 | 1.74 | 111.8 | 154.5 | 3.99 |
| Patient-144 | LUSC | Male | 68 | Yes | No | 4 | 3 | 0 | IIIC | 22.55 | 79.13 | 60.6 | 6.75 | 110.5 | 196.0 | 93.99 |
| Patient-145 | LUSC | Male | 82 | Yes | No | 2b | x | 1 | IV | 19.00 | 93.16 | 48.1 | 6.61 | 111.1 | 222.7 | 3.77 |
| Patient-146 | LUAD | Female | 75 | No | No | 4 | 3 | 1 | IV | 20.21 | 147.37 | 60.6 | 2.30 | 190.2 | 169.3 | 4.64 |
| Patient-147 | LUAD | Female | 68 | No | No | 2a | x | 1b | IVa | 10.24 | 161.40 | 152.5 | 4.25 | 191.7 | 184.2 | 8.51 |
| Patient-148 | LUAD | Male | 58 | Yes | No | 1c | 2 | 0 | IIIA | 9.44 | 153.78 | 76.3 | 2.85 | 115.0 | 154.5 | 2.48 |
| Patient-149 | LUAD | Male | 70 | Yes | Yes | 2a | 1 | 0 | IIB | 12.98 | 37.36 | 36.7 | 8.97 | 107.8 | 110.1 | 52.77 |
| Patient-150 | LUAD | Female | 55 | No | No | 1 | 1 | 0 | IIB | 20.41 | 78.08 | 39.6 | 12.31 | 109.2 | 157.5 | NA |
| Patient-151 | LUAD | Male | 60 | No | No | 4 | 1 | 0 | IIIA | 14.79 | 71.52 | 46.2 | 15.65 | 105.8 | 197.5 | 2.23 |
| Patient-152 | LUAD | Female | 48 | No | No | 2 | 0 | 1c | IVB | 12.82 | 72.05 | 108.6 | 14.82 | 191.2 | 160.5 | 41.05 |
| Patient-153 | SCLC | Male | 64 | Yes | Yes | 1c | 2 | 1c | IVB | 13.12 | 70.92 | 126.0 | 9.25 | 252.7 | 200.5 | NA |
| Patient-154 | LUSC | Male | 81 | Yes | No | 4 | 3 | 1 | IV | 5.69 | 37.36 | 126.0 | 5.08 | 252.0 | 199.0 | 2.35 |
| Patient-155 | LUAD | Female | 68 | Yes | No | 2a | 3 | 1b | IV | 2.01 | 222.39 | 343.7 | 7.31 | 195.8 | 219.7 | 6.89 |
| Patient-156 | NSCLC | Male | 68 | NA | NA | 1b | 2 | 0 | IIIA | 6.86 | 153.48 | 107.9 | 3.41 | 110.5 | 169.3 | NA |
| Patient-157 | LUSC | Male | 75 | Yes | Yes | 3 | 3 | 0 | IIIB | 4.79 | 78.38 | 88.4 | 8.70 | 105.8 | 185.6 | 3.91 |
| Patient-158 | LUAD | Male | 60 | No | No | 4 | 3 | 1b | IVb | 7.00 | 71.52 | 296.8 | 8.28 | 191.2 | 216.8 | 8.84 |
| Patient-159 | LUSC | Female | 66 | No | No | 4 | 3 | 0 | IIIB | 5.49 | 35.10 | 76.3 | 9.39 | 104.5 | 228.6 | 35.6 |
| Patient-160 | LUSC | Male | 87 | No | No | 3 | 1 | 0 | IIIA | 8.77 | 56.51 | 92.7 | 9.53 | 107.2 | 199.0 | 21.91 |
| Patient-161 | LUAD | Female | 68 | No | No | 1c | 1 | 0 | IIB | 9.17 | 71.37 | 31.5 | 9.95 | 110.5 | 121.9 | 4.36 |
| Patient-162 | LUAD | Female | 52 | No | No | 4 | 3 | 1c | IVb | 9.84 | 63.30 | 196.8 | 11.90 | 193.8 | 255.3 | 183.4 |
| Patient-163 | LUAD | Male | 82 | Yes | Yes | x | x | 1 | IV | 13.12 | 78.31 | 358.6 | 21.08 | 194.3 | 224.2 | 25.76 |
| Patient-164 | LUAD | Female | 66 | No | No | 1c | 0 | 0 | IA3 | 12.25 | 82.45 | 74.3 | 3.69 | 7.3 | 127.9 | NA |
| Patient-165 | LUSC | Male | 60 | Yes | No | 4 | 3 | 1 | IV | 11.31 | 153.78 | 258.5 | 12.87 | 168.8 | 154.5 | 4.34 |
| Patient-166 | LUAD | Female | 69 | No | No | 1c | 0 | 0 | IA3 | 15.66 | 36.68 | 16.0 | 7.86 | 98.5 | 127.9 | NA |
| Patient-167 | LUSC | Male | 59 | No | No | 3 | 0 | 0 | IIB | 12.05 | 51.69 | 51.1 | 6.19 | 111.8 | 139.7 | 4.33 |
| Patient-168 | LUAD | Male | 75 | Yes | No | 1c | 0 | 0 | IA3 | 12.38 | 34.35 | 40.0 | 2.30 | 109.2 | 154.5 | NA |
| Patient-169 | LUAD | Male | 69 | No | No | 1c | 0 | 0 | IA | 12.15 | 45.58 | 73.7 | 5.64 | 189.7 | 107.1 | 0.693 |
| Patient-170 | LUAD | Female | 45 | No | Yes | 1b | 1 | 0 | IIB | 12.85 | 65.56 | 87.0 | 7.03 | 192.8 | 190.1 | NA |
| Patient-171 | LUAD | Male | 70 | Yes | Yes | 3 | 1 | 0 | IIIA | 24.49 | 57.19 | 106.3 | 5.08 | 107.2 | 222.7 | 44.15 |
| Patient-172 | NSCLC | Male | 79 | Yes | No | 1c | 3 | 0 | IIIB | 24.42 | 51.76 | 167.2 | 3.69 | 188.7 | 196.0 | 14.38 |
| Patient-173 | NSCLC | Male | 54 | No | Yes | 1b | 1 | 0 | IIB | 29.24 | 73.03 | 26.4 | 1.19 | 113.1 | 121.9 | NA |
| Patient-174 | SCLC | Male | 55 | Yes | Yes | 4 | 1 | 0 | IIIA | 16.43 | 81.92 | 89.1 | 4.52 | 112.4 | 293.8 | 20.15 |
| Patient-175 | LUAD | Female | 30 | No | No | is | 0 | 0 | Tis | 19.74 | 84.79 | 89.1 | 4.38 | 114.4 | 99.1 | NA |
| Patient-176 | LUSC | Male | 60 | Yes | Yes | 3 | 2 | 0 | IIIB | 18.77 | 93.54 | 147.7 | 12.45 | 193.3 | 874.7 | 4.48 |
| Patient-177 | LUAD | Female | 78 | No | No | 3 | 3 | 1a | IVA | 17.16 | 79.89 | 20.7 | 9.81 | 111.8 | 191.6 | NA |
| Patient-178 | LUAD | Female | 64 | No | No | 1mi | 1 | 0 | IIB | 11.38 | 72.35 | 76.9 | 8.84 | 109.8 | 151.6 | NA |
| Patient-179 | LUAD | Female | 60 | No | No | 1b | 0 | 0 | IA2 | 21.21 | 82.90 | 53.6 | 1.19 | 105.2 | 124.9 | NA |
| Patient-180 | LUAD | Female | 41 | No | Yes | 1mi | 1 | 0 | IIB | 16.93 | 71.67 | 258.5 | 2.02 | 101.2 | 196.0 | NA |
| Patient-181 | LUAD | Male | 70 | Yes | Yes | 2a | 0 | 0 | IB | 0.67 | 76.42 | 106.3 | 8.14 | 93.0 | 124.9 | NA |
| Patient-182 | SCLC | Male | 51 | Yes | Yes | 4b | 3 | 1a | IVA | 5.72 | 71.67 | 295.4 | 6.47 | 253.5 | 329.4 | 5.56 |
| Patient-183 | LUAD | Female | 56 | No | No | 1b | 1 | 0 | IIB | 8.30 | 64.81 | 50.1 | 2.85 | 191.7 | 190.1 | 2.61 |
| Patient-184 | LUAD | Male | 62 | No | No | 1b | 1 | 0 | IIB | 5.56 | 56.59 | 73.7 | 4.94 | 102.5 | 188.6 | NA |
| Patient-185 | LUAD | Male | 60 | Yes | No | 1c | 0 | 0 | IA3 | 24.36 | 72.27 | 62.9 | 3.41 | 98.5 | 113.0 | 4.22 |
| Patient-186 | LUAD | Female | 60 | No | No | tis | 0 | 0 | 0 | 17.67 | 71.90 | 18.2 | 5.22 | 87.5 | 127.9 | NA |
| Patient-187 | LUAD | Female | 51 | No | No | 1c | 1 | 0 | IIB | 19.67 | 70.77 | 52.1 | 4.80 | 91.6 | 154.5 | **NA** |
| Patient-188 | NSCLC | Male | 72 | Yes | Yes | 3 | 2 | 0 | IIIB | 17.20 | 64.05 | 107.9 | 9.81 | 191.2 | 282.0 | NA |
| Patient-189 | LUAD | Male | 67 | Yes | Yes | 4 | 3 | 1 | IV | 17.26 | 64.51 | 329.1 | 2.30 | 314.8 | 459.8 | 0.846 |
| Patient-190 | NSCLC | Male | 52 | Yes | No | 4 | 3 | 0 | IIIC | 17.03 | 66.32 | 145.8 | 3.83 | 68.4 | 219.7 | 7.57 |
| Patient-191 | LUAD | Male | 38 | No | No | 1a | 1 | 0 | IIB | 16.96 | 61.42 | 62.9 | 4.38 | 58.2 | 127.9 | NA |
| Patient-192 | LUAD | Female | 23 | No | No | 1a | 1 | 0 | IIB | 17.33 | 71.59 | 52.1 | 5.64 | 70.6 | 157.5 | NA |
| Patient-193 | LUAD | Male | 56 | No | No | 4 | 2 | 0 | IIIB | 17.60 | 75.06 | 106.3 | 6.75 | 236.4 | 190.1 | NA |
| Patient-194 | LUAD | Male | 67 | Yes | Yes | 2 | 2 | 1 | IV | 4.69 | 64.66 | 511.5 | 13.43 | 252.0 | 284.9 | NA |
| Patient-195 | NSCLC | Male | 80 | No | No | 3 | 2 | 0 | IIIB | 3.42 | 57.95 | 24.7 | 7.03 | 192.3 | 200.5 | 2.5 |
| Patient-196 | LUAD | Male | 71 | No | Yes | 2a | 2 | 1c | IVb | 14.66 | 71.97 | 458.6 | 3.97 | 166.5 | 156.0 | 8.58 |
| Patient-197 | LUSC | Male | 62 | Yes | Yes | 4 | 2 | 1a | IVA | 12.78 | 64.66 | 166.2 | 4.66 | 300.5 | 332.3 | 4.22 |
| Patient-198 | LUAD | Female | 41 | No | No | 1a | 1 | 0 | IIB | 13.15 | 70.77 | 37.1 | 12.03 | 95.1 | 160.5 | NA |
| Patient-199 | SCLC | Female | 74 | No | No | 4 | 2 | 1c | IVB | 13.58 | 48.60 | 169.2 | 12.45 | 202.3 | 282.0 | 5 |
| Patient-200 | LUSC | Male | 67 | Yes | Yes | 2 | 2 | 0 | IIIA | 5.56 | 45.20 | 126.0 | 5.64 | 195.3 | 187.1 | 2.51 |
| Patient-201 | LUAD | Female | 71 | No | No | 1 | 0 | 0 | IA2 | 22.08 | 59.30 | 19.4 | 3.83 | 98.5 | 124.9 | NA |
| Patient-202 | LUSC | Male | 69 | Yes | Yes | 4 | 2 | 0 | IIIB | 20.21 | 55.46 | 36.7 | 2.30 | 285.1 | 110.1 | NA |
| Patient-203 | LUAD | Female | 32 | No | No | 1a | 1 | 0 | IIB | 4.29 | 66.09 | 39.2 | 2.58 | 83.3 | 190.1 | NA |
| Patient-204 | LUSC | Male | 56 | Yes | No | 1 | 2 | 0 | IIIA | 17.26 | 82.38 | 62.9 | 3.41 | 190.2 | 225.7 | 2.07 |
| Patient-205 | LUAD | Male | 72 | No | No | 2a | 3 | 1b | IV | 16.06 | 78.68 | 175.4 | 1.46 | 249.8 | 222.7 | 4.52 |
| Patient-206 | LUSC | Male | 58 | Yes | Yes | 4 | 2 | 0 | IIIB | 18.87 | 77.85 | 51.1 | 2.58 | 294.9 | 286.4 | 2.79 |
| Patient-207 | LUAD | Female | 60 | No | No | 2a | 0 | 0 | IB | 2.11 | 52.44 | 41.7 | 2.02 | 74.8 | 133.8 | **NA** |
| Patient-208 | LUSC | Male | 58 | Yes | No | 2 | 2 | 0 | IIIA | 12.31 | 48.30 | 108.7 | 1.74 | 178.6 | 221.2 | 3.21 |
| Patient-209 | LUAD | Female | 61 | No | No | 4 | 3 | 1c | IVB | 9.87 | 44.83 | 191.3 | 4.38 | 115.7 | 196.0 | NA |
| Patient-210 | LUAD | Male | 56 | Yes | No | 1b | 0 | 0 | IA2 | 5.59 | 37.44 | 31.5 | 7.31 | 84.7 | 187.1 | NA |
| Patient-211 | LUSC | Male | 67 | Yes | No | 3 | 0 | 0 | IIB | 19.34 | 33.22 | 40.4 | 9.67 | 114.4 | 194.5 | 2.58 |
| Patient-212 | LUSC | Male | 56 | Yes | Yes | 1c | 1 | 1a | IVA | 6.83 | 33.59 | 19.8 | 5.36 | 102.5 | 130.8 | **NA** |
| Patient-213 | LUAD | Female | 53 | No | No | 1 | 1 | 0 | IIB | 6.03 | 44.83 | 32.2 | 3.97 | 153.9 | 124.9 | **NA** |
| Patient-214 | LUAD | Female | 55 | No | No | 1c | 1 | 0 | IIB | 23.35 | 59.53 | 33.7 | 1.19 | 128.5 | 139.7 | **1.78** |
| Patient-215 | NSCLC | Male | 66 | Yes | No | 4 | 2 | 0 | IIIB | 11.31 | 79.13 | 50.1 | 1.05 | 199.3 | 154.5 | 4.04 |
| Patient-216 | SCLC | Female | 66 | Yes | No | 2b | 3 | 1c | IVB | 10.34 | 75.67 | 168.2 | 15.51 | 202.3 | 204.9 | 4.38 |
| Patient-217 | LUSC | Male | 65 | Yes | Yes | 3 | 2 | 1a | IIIB | 16.53 | 51.84 | 40.0 | 14.82 | 113.7 | 164.9 | 6.32 |
| Patient-218 | LUAD | Male | 75 | Yes | Yes | 1b | 0 | 1a | IVA | 11.44 | 70.61 | 115.9 | 2.44 | 129.7 | 219.7 | 22.69 |
| Patient-219 | LUSC | Male | 65 | Yes | Yes | 4 | 2 | 1b | IVA | 4.95 | 63.07 | 472.4 | 15.10 | 155.1 | 193.1 | NA |
| Patient-220 | LUSC | Male | 54 | Yes | No | 2a | 0 | 0 | IB | 4.82 | 79.89 | 337.8 | 5.91 | 84.0 | 145.6 | NA |
| Patient-221 | LUAD | Female | 63 | No | No | 2a | 2 | 0 | IIIA | 4.69 | 73.10 | 50.1 | 1.46 | 166.5 | 148.6 | NA |
| Patient-222 | SCLC | Male | 52 | Yes | Yes | 3 | 3 | 1C | IVB | 4.62 | 55.01 | 292.7 | 2.72 | 205.2 | 261.2 | 10.81 |
| Patient-223 | LUAD | Male | 40 | Yes | Yes | 3 | 2 | 1C | IVB | 17.40 | 33.14 | 115.9 | 3.97 | 260.5 | 210.8 | 99.25 |
| Patient-224 | LUAD | Male | 44 | No | No | 3 | 2 | 1a | IVA | 11.58 | 37.44 | 59.5 | 4.25 | 248.6 | 166.4 | NA |
| Patient-225 | LUAD | Female | 35 | No | No | 2b | 2 | 0 | IIIA | 13.52 | 51.61 | 76.3 | 5.78 | 98.5 | 145.6 | **NA** |
| Patient-226 | LUAD | Female | 59 | No | No | 1c | 2 | 1a | IV | 12.72 | 53.95 | 192.4 | 3.69 | 109.8 | 199.0 | 12.57 |
| Patient-227 | LUAD | Male | 58 | No | No | 1b | 3 | 1c | IVB | 12.35 | 63.90 | 98.6 | 3.13 | 85.4 | 234.5 | **48.23** |
| Patient-228 | LUAD | Female | 68 | No | No | 1b | 0 | 1c | IVB | 4.79 | 67.30 | 61.7 | 1.19 | 140.2 | 199.0 | NA |
| Patient-229 | LUSC | Male | 59 | Yes | No | 1b | 0 | 0 | IA2 | 6.86 | 29.75 | 34.4 | 0.35 | 86.1 | 184.2 | NA |
| Patient-230 | LUAD | Male | 73 | No | No | 1c | 0 | 0 | IA3 | 5.62 | 105.15 | 61.7 | 2.72 | 95.7 | 107.1 | 2.91 |
| Patient-231 | LUAD | Male | 53 | No | No | 1b | 0 | 0 | IA2 | 17.33 | 44.75 | 75.6 | 4.25 | 137.8 | 199.0 | **NA** |
| Patient-232 | LUAD | Female | 65 | No | No | 1b | 0 | 0 | IA2 | 20.21 | 44.53 | 39.2 | 4.52 | 192.3 | 124.9 | **NA** |
| Patient-233 | LUAD | Male | 69 | Yes | No | 2 | 2 | 0 | IIIA | 4.75 | 52.37 | 62.9 | 5.78 | 212.8 | 107.1 | **13.44** |
| Patient-234 | LUAD | Male | 67 | No | No | 2a | 1 | 0 | IIB | 6.03 | 32.84 | 88.4 | 4.80 | 114.4 | 160.5 | 9.01 |
| Patient-235 | LUSC | Female | 51 | No | No | 2a | 2 | 0 | IIIA | 8.30 | 16.70 | 75.0 | 6.61 | 118.3 | 110.1 | 1.16 |
| Patient-236 | LUAD | Female | 72 | No | No | 1c | 1 | 0 | II | 8.70 | 12.10 | 61.7 | 2.69 | 124.7 | 110.1 | NA |
| Patient-237 | LUSC | Male | 57 | Yes | Yes | 4 | 3 | 0 | IIIC | 9.04 | 79.89 | 75.6 | 1.60 | 167.7 | 196.0 | 2.81 |
| Patient-238 | LUSC | Male | 70 | No | No | 4 | 2 | 0 | IIIC | 21.41 | 70.77 | 62.9 | 9.53 | 179.7 | 284.9 | NA |
| Patient-239 | NSCLC | Male | 68 | No | No | 1c | 0 | 0 | IA3 | 12.31 | 72.35 | 70.6 | 0.49 | 129.7 | 187.1 | NA |
| Patient-240 | LUAD | Male | 69 | Yes | Yes | 2b | 0 | 1b | IVA | 19.07 | 40.53 | 126.9 | 5.50 | 223.7 | 1025.8 | 162.1 |
| Patient-241 | LUSC | Female | 57 | No | No | 2a | 1 | 0 | IIB | 18.77 | 59.23 | 32.2 | 0.91 | 115.0 | 124.9 | NA |
| Patient-242 | LUSC | Female | 63 | No | No | 3 | 0 | 0 | IIB | 4.65 | 57.80 | 50.1 | 7.17 | 127.8 | 169.3 | 1.94 |
| Patient-243 | LUAD | Female | 60 | No | No | tis | 0 | 0 | 0 | 4.89 | 22.36 | 25.5 | 6.75 | 84.7 | 104.1 | NA |
| Patient-244 | LUAD | Female | 68 | No | No | 1b | 0 | 0 | IA2 | 3.88 | 33.59 | 241.0 | 4.52 | 74.8 | 107.1 | NA |
| Patient-245 | LUAD | Male | 65 | Yes | Yes | 1b | 1 | 0 | IIB | 21.41 | 50.86 | 41.7 | 5.64 | 127.2 | 110.1 | NA |
| Patient-246 | LUAD | Female | 66 | Yes | No | 2a | 2 | 1c | IVB | 4.89 | 60.13 | 175.4 | 0.91 | 242.9 | 302.7 | 9.21 |
| Patient-247 | LUAD | Male | 58 | Yes | No | 3 | 2 | 0 | IIIB | 0.07 | 74.69 | 73.7 | 0.63 | 194.3 | 193.1 | **20.21** |
| Patient-248 | LUAD | Male | 60 | Yes | No | 4 | 3 | 0 | IIIC | 2.21 | 79.59 | 92.7 | 0.77 | 103.8 | 204.9 | 2.22 |
| Patient-249 | LUAD | Female | 70 | No | No | 2b | 1 | 0 | IIB | 2.48 | 85.92 | 50.1 | 1.19 | 98.5 | 121.9 | 17.53 |
| Patient-250 | LUAD | Male | 65 | No | No | 1b | 0 | 0 | IA2 | 5.62 | 160.79 | 39.2 | 1.74 | 69.1 | 80.4 | NA |
| Patient-251 | LUSC | Male | 73 | Yes | No | 1b | 0 | 0 | IA2 | 3.08 | 86.00 | 36.7 | 1.46 | 114.4 | 107.1 | NA |
| Patient-252 | LUSC | Male | 82 | Yes | No | 1c | 3 | 0 | IIIB | 3.15 | 59.30 | 41.7 | 1.60 | 178.1 | 323.5 | NA |
| Patient-253 | LUSC | Male | 84 | Yes | No | 4 | 3 | 1c | IVB | 4.89 | 82.15 | 107.9 | 4.38 | 285.6 | 453.9 | 10.52 |
| Patient-254 | LUAD | Female | 69 | No | No | 1c | 0 | 0 | IA3 | 5.46 | 78.61 | 44.4 | 6.19 | 79.1 | 68.6 | NA |
| Patient-255 | LUSC | Male | 58 | Yes | No | 1b | 1 | 0 | IIB | 4.65 | 58.48 | 51.1 | 8.00 | 61.9 | 101.2 | NA |
| Patient-256 | LUAD | Male | 65 | Yes | No | 3 | 2 | 0 | IIIB | 5.56 | 82.53 | 75.0 | 9.53 | 214.2 | 154.5 | 14.1 |
| Patient-257 | SCLC | Male | 72 | Yes | Yes | 2b | 2 | 1c | IVB | 11.91 | 93.54 | 194.6 | 4.11 | 241.7 | 548.7 | 15.78 |
| Patient-258 | SCLC | Male | 63 | Yes | Yes | X | X | 1c | IVB | 11.31 | 105.22 | 145.8 | 9.81 | 224.6 | 224.2 | 13.94 |
| Control-1 | Con | Male | 69 | No | No | NA | NA | NA | NA | 2.58 | 50.97 | 22.38 | 10.69 | 34.51 | 88.69 | 1.971 |
| Control-2 | Con | Female | 67 | No | No | NA | NA | NA | NA | 1.99 | 37.41 | 12.38 | 9.12 | 45.42 | 56.59 | 2.606 |
| Control-3 | Con | Female | 66 | No | No | NA | NA | NA | NA | 3.12 | 42.81 | 5.69 | 11.61 | 23.41 | 78.79 | 0.675 |
| Control-4 | Con | Male | 66 | Yes | Yes | NA | NA | NA | NA | 2.01 | 51.41 | 9.95 | 9.91 | 16.40 | 81.62 | 3.459 |
| Control-5 | Con | Male | 66 | Yes | Yes | NA | NA | NA | NA | 15.53 | 67.28 | 15.74 | 10.59 | 18.48 | 361.02 | 5.56 |
| Control-6 | Con | Male | 65 | Yes | Yes | NA | NA | NA | NA | 4.01 | 76.70 | 19.92 | 8.59 | 166.59 | 39.49 | 2.626 |
| Control-7 | Con | Male | 65 | No | No | NA | NA | NA | NA | 3.78 | 79.17 | 51.29 | 11.79 | 170.59 | 45.56 | 5.4883 |
| Control-8 | Con | Male | 63 | Yes | Yes | NA | NA | NA | NA | 4.52 | 53.05 | 191.28 | 17.45 | 12.39 | 36.48 | 3.458 |
| Control-9 | Con | Male | 62 | No | Yes | NA | NA | NA | NA | 2.77 | 17.89 | 34.59 | 9.17 | 14.58 | 38.95 | 2.369 |
| Control-10 | Con | Female | 62 | No | No | NA | NA | NA | NA | 3.22 | 82.30 | 29.93 | 17.87 | 158.59 | 57.68 | 1.9 |
| Control-11 | Con | Female | 61 | No | No | NA | NA | NA | NA | 1.96 | 33.54 | 31.29 | 8.69 | 34.59 | 78.38 | 2.808 |
| Control-12 | Con | Female | 61 | No | No | NA | NA | NA | NA | 1.07 | 38.95 | 81.29 | 8.41 | 56.42 | 56.47 | 0.83 |
| Control-13 | Con | Female | 61 | No | No | NA | NA | NA | NA | 19.07 | 102.35 | 22.39 | 11.69 | 211.29 | 142.33 | 2.885 |
| Control-14 | Con | Male | 60 | No | No | NA | NA | NA | NA | 2.11 | 60.04 | 5.69 | 7.55 | 24.57 | 38.49 | 1.704 |
| Control-15 | Con | Female | 59 | No | No | NA | NA | NA | NA | 3.67 | 38.66 | 9.84 | 10.39 | 34.32 | 39.45 | 2.52 |
| Control-16 | Con | Female | 59 | No | No | NA | NA | NA | NA | 3.51 | 47.27 | 8.48 | 8.57 | 26.47 | 59.29 | 0.79 |
| Control-17 | Con | Male | 77 | Yes | Yes | NA | NA | NA | NA | 1.98 | 19.42 | 18.80 | 30.81 | 48.24 | 134.51 | 4.481 |
| Control-18 | Con | Male | 71 | Yes | No | NA | NA | NA | NA | 0.17 | 25.68 | 50.06 | 14.68 | 23.48 | 61.11 | 2.369 |
| Control-19 | Con | Female | 69 | No | No | NA | NA | NA | NA | 2.58 | 37.36 | 23.07 | 21.08 | 33.43 | 90.61 | 2.922 |
| Control-20 | Con | Male | 62 | No | No | NA | NA | NA | NA | 5.82 | 36.16 | 30.17 | 17.60 | 28.04 | 74.64 | 2.8405 |
| Control-21 | Con | Male | 59 | Yes | Yes | NA | NA | NA | NA | 2.38 | 39.21 | 30.85 | 19.96 | 31.71 | 85.51 | 1.51 |
| Control-22 | Con | Male | 57 | Yes | No | NA | NA | NA | NA | 0.37 | 36.04 | 17.36 | 23.44 | 37.06 | 101.38 | 6.003 |
| Control-23 | Con | Female | 57 | No | Yes | NA | NA | NA | NA | 2.28 | 45.43 | 15.60 | 13.98 | 22.39 | 57.88 | 4.422 |
| Control-24 | Con | Male | 56 | No | Yes | NA | NA | NA | NA | 1.58 | 49.28 | 23.33 | 6.61 | 10.69 | 23.20 | 2.469 |
| Control-25 | Con | Male | 56 | Yes | Yes | NA | NA | NA | NA | 2.31 | 52.97 | 108.65 | 10.09 | 16.23 | 39.64 | 3.721 |
| Control-26 | Con | Female | 56 | No | No | NA | NA | NA | NA | 1.68 | 29.90 | 28.85 | 39.44 | 61.03 | 172.41 | 0.576 |
| Control-27 | Con | Male | 68 | No | No | NA | NA | NA | NA | 4.39 | 33.29 | 26.38 | 14.54 | 23.26 | 60.47 | 2.7 |
| Control-28 | Con | Female | 55 | No | No | NA | NA | NA | NA | 2.41 | 61.04 | 27.59 | 12.31 | 19.76 | 50.08 | 1.491 |
| Control-29 | Con | Female | 55 | No | No | NA | NA | NA | NA | 2.81 | 59.87 | 24.67 | 76.86 | 112.90 | 326.13 | 1.209 |
| Control-30 | Con | Female | 55 | No | No | NA | NA | NA | NA | 7.66 | 58.25 | 30.85 | 9.25 | 14.91 | 35.71 | 3.101 |
| Control-31 | Con | Female | 55 | No | No | NA | NA | NA | NA | 9.40 | 35.89 | 20.68 | 10.64 | 17.12 | 42.26 | 4.082 |
| Control-32 | Con | Male | 69 | Yes | Yes | NA | NA | NA | NA | 10.37 | 84.64 | 19.60 | 12.73 | 20.42 | 52.03 | 0.549 |
| Control-33 | Con | Male | 55 | No | Yes | NA | NA | NA | NA | 7.63 | 75.74 | 21.36 | 37.07 | 57.56 | 162.11 | 1.92 |
| Control-34 | Con | Male | 59 | No | No | NA | NA | NA | NA | 8.70 | 80.57 | 24.39 | 11.48 | 18.44 | 46.17 | NA |
| Control-35 | Con | Female | 55 | No | No | NA | NA | NA | NA | 12.38 | 71.33 | 23.07 | 8.56 | 13.80 | 32.42 | 0.89 |
| Control-36 | Con | Female | 55 | Yes | No | NA | NA | NA | NA | 8.03 | 40.19 | 38.34 | 11.90 | 19.10 | 48.13 | 2.539 |
| Control-37 | Con | Female | 54 | No | No | NA | NA | NA | NA | 1.94 | 16.85 | 49.08 | 1.32 | 2.16 | -2.08 | 1.349 |
| Control-38 | Con | Female | 54 | No | No | NA | NA | NA | NA | 1.74 | 30.12 | 15.23 | 29.28 | 45.94 | 127.69 | 2.1 |
| Control-39 | Con | Male | 62 | Yes | No | NA | NA | NA | NA | 3.82 | 36.61 | 52.57 | 12.31 | 19.76 | 50.08 | 7.35 |
| Control-40 | Con | Male | 58 | Yes | Yes | NA | NA | NA | NA | 5.66 | 42.87 | 16.42 | 15.79 | 25.22 | 66.28 | 8.18 |
| Control-41 | Con | Male | 53 | Yes | No | NA | NA | NA | NA | 3.12 | 37.97 | 15.86 | 14.96 | 23.91 | 62.40 | 1.99 |
| Control-42 | Con | Male | 53 | Yes | No | NA | NA | NA | NA | 2.48 | 37.66 | 19.81 | 11.48 | 18.44 | 46.17 | 1.166 |
| Control-43 | Con | Male | 64 | Yes | Yes | NA | NA | NA | NA | 2.31 | 53.01 | 25.23 | 19.69 | 31.28 | 84.24 | 1.35 |
| Control-44 | Con | Female | 53 | No | No | NA | NA | NA | NA | 4.42 | 52.03 | 21.60 | 8.97 | 14.46 | 34.39 | 0.288 |
| Control-45 | Con | Male | 60 | No | No | NA | NA | NA | NA | 5.42 | 51.46 | 16.72 | 43.19 | 66.50 | 188.62 | 2.5866 |
| Control-46 | Con | Female | 52 | No | No | NA | NA | NA | NA | 6.36 | 29.75 | 15.86 | 0.91 | 1.48 | -4.09 | 1.871 |
| Control-47 | Con | Male | 52 | No | Yes | NA | NA | NA | NA | 2.01 | 34.65 | 45.29 | 15.51 | 24.79 | 64.99 | 3.644 |
| Control-48 | Con | Male | 51 | Yes | Yes | NA | NA | NA | NA | 5.72 | 38.68 | 23.59 | 12.45 | 19.98 | 50.73 | 1.306 |
| Control-49 | Con | Male | 68 | Yes | No | NA | NA | NA | NA | 1.31 | 37.97 | 50.06 | 16.21 | 25.87 | 68.21 | 4.986 |
| Control-50 | Con | Female | 51 | No | No | NA | NA | NA | NA | 2.75 | 46.94 | 31.54 | 14.96 | 23.91 | 62.40 | 1.9 |
| Control-51 | Con | Female | 74 | No | No | NA | NA | NA | NA | 2.38 | 37.78 | 28.53 | 8.14 | 13.13 | 30.45 | 1.377 |
| Control-52 | Con | Male | 51 | No | Yes | NA | NA | NA | NA | 2.30 | 15.27 | 28.85 | 9.25 | 14.91 | 35.71 | 0.523 |
| Control-53 | Con | Female | 51 | No | No | NA | NA | NA | NA | 2.58 | 60.32 | 35.19 | 65.17 | 97.34 | 280.00 | 0.32 |
| Control-54 | Con | Male | 50 | Yes | No | NA | NA | NA | NA | 5.69 | 71.22 | 21.83 | 0.91 | 1.48 | -4.09 | 2.093 |
| Control-55 | Con | Male | 65 | Yes | No | NA | NA | NA | NA | 6.09 | 42.94 | 19.81 | 2.85 | 4.64 | 5.27 | 2.626 |
| Control-56 | Con | Male | 62 | Yes | No | NA | NA | NA | NA | 4.92 | 44.68 | 19.40 | 6.47 | 10.46 | 22.54 | 0.477 |
| Control-57 | Con | Female | 50 | No | Yes | NA | NA | NA | NA | 4.55 | 45.51 | 22.82 | 6.33 | 10.24 | 21.88 | 1.749 |
| Control-58 | Con | Female | 68 | No | No | NA | NA | NA | NA | 7.70 | 55.61 | 18.24 | 5.78 | 9.35 | 19.23 | 1.22 |
| Control-59 | Con | Male | 50 | Yes | No | NA | NA | NA | NA | 2.04 | 50.29 | 40.85 | 9.11 | 14.68 | 35.05 | ＜0.2 |
| Control-60 | Con | Male | 70 | Yes | No | NA | NA | NA | NA | 3.42 | 47.69 | 23.07 | 14.96 | 23.91 | 62.40 | 1.6641 |
| Control-61 | Con | Male | 50 | No | No | NA | NA | NA | NA | 3.82 | 34.05 | 17.70 | 11.34 | 18.22 | 45.52 | 0.79 |
| Control-62 | Con | Male | 63 | Yes | Yes | NA | NA | NA | NA | 7.10 | 37.36 | 35.95 | 3.69 | 5.99 | 9.27 | 2.822 |
| Control-63 | Con | Female | 69 | Yes | No | NA | NA | NA | NA | 1.38 | 57.08 | 18.42 | 40.69 | 62.86 | 177.83 | 3.745 |
| Control-64 | Con | Male | 49 | Yes | No | NA | NA | NA | NA | 5.76 | 60.70 | 19.00 | 8.70 | 14.02 | 33.08 | 3.128 |
| Control-65 | Con | Male | 58 | Yes | No | NA | NA | NA | NA | 1.74 | 30.12 | 20.68 | 6.61 | 10.69 | 23.20 | 5.68 |
| Control-66 | Con | Male | 63 | No | No | NA | NA | NA | NA | 5.42 | 41.43 | 16.57 | 31.37 | 49.08 | 136.98 | 0.8757 |
| Control-67 | Con | Male | 66 | Yes | No | NA | NA | NA | NA | 4.99 | 45.62 | 18.61 | 35.54 | 55.30 | 155.41 | 2.372 |
| Control-68 | Con | Male | 47 | Yes | Yes | NA | NA | NA | NA | 8.43 | 47.92 | 17.03 | 30.40 | 47.62 | 132.65 | 3.358 |
| Control-69 | Con | Male | 74 | No | No | NA | NA | NA | NA | 10.04 | 52.97 | 51.06 | 31.79 | 49.70 | 138.83 | 1.652 |
| Control-70 | Con | Female | 73 | No | No | NA | NA | NA | NA | 1.64 | 52.82 | 94.16 | 56.27 | 85.09 | 243.71 | 1.6893 |
| Control-71 | Con | Male | 69 | Yes | No | NA | NA | NA | NA | 5.02 | 36.68 | 15.60 | 43.19 | 66.50 | 188.62 | 3.5923 |
| Control-72 | Con | Male | 65 | Yes | No | NA | NA | NA | NA | 17.26 | 41.36 | 29.50 | 31.23 | 48.87 | 136.36 | 2.093 |
| Control-73 | Con | Male | 68 | Yes | No | NA | NA | NA | NA | 6.29 | 43.24 | 19.00 | 42.64 | 65.70 | 186.23 | 2.973 |
| Control-74 | Con | Female | 60 | No | No | NA | NA | NA | NA | 10.07 | 36.91 | 20.02 | 30.95 | 48.45 | 135.13 | 1.1543 |
| Control-75 | Con | Male | 59 | Yes | No | NA | NA | NA | NA | 9.07 | 37.06 | 16.13 | 14.96 | 23.91 | 62.40 | 2.1328 |
| Control-76 | Con | Male | 70 | Yes | Yes | NA | NA | NA | NA | 2.38 | 52.44 | 24.12 | 10.37 | 16.67 | 40.95 | 3.847 |
| Control-77 | Con | Male | 79 | Yes | No | NA | NA | NA | NA | 5.62 | 54.40 | 18.24 | 19.13 | 30.42 | 81.68 | 3.853 |
| Control-78 | Con | Male | 83 | Yes | No | NA | NA | NA | NA | 7.03 | 79.81 | 41.71 | 34.01 | 53.02 | 148.68 | 3.7902 |
| Control-79 | Con | Male | 56 | Yes | No | NA | NA | NA | NA | 8.30 | 86.00 | 16.27 | 30.95 | 48.45 | 135.13 | 1.384 |
| Control-80 | Con | Female | 64 | Yes | No | NA | NA | NA | NA | 5.09 | 78.08 | 34.43 | 39.58 | 61.24 | 173.01 | 1.326 |
